# Using T‐cell repertoire profiles as predictor in a primary mucosal melanoma

**DOI:** 10.1002/ctm2.136

**Published:** 2020-08-11

**Authors:** Che‐Mai Chang, Yu‐Ming Liao, Gong‐Yau Lan, Wei‐Chiao Chang, Yun Yen

**Affiliations:** ^1^ Ph.D. Program in Medical Biotechnology, College of Medical Science and Technology Taipei Medical University Taipei Taiwan; ^2^ Ph.D. Program for Translational Medicine, College of Medical Science and Technology Taipei Medical University and Academia Sinica Taipei Taiwan; ^3^ Section of Diagnostic Radiology, Department of Medical Imaging Taipei Medical University Hospital Taipei Taiwan; ^4^ Department of Clinical Pharmacy, School of Pharmacy Taipei Medical University Taipei Taiwan; ^5^ Integrative Therapy Center for Gastroenterologic Cancers, Wan Fang Hospital Taipei Medical University Taipei Taiwan; ^6^ Ph.D. Program for Cancer Molecular Biology and Drug Discovery, College of Medical Science and Technology Taipei Medical University Taipei Taiwan; ^7^ Division of Chemistry and Chemical Engineering California Institute of Technology Pasadena California

Dear Editor,

Primary mucosal melanoma is a rare subtype of melanoma that carries poor prognosis. Traditional treatment options of mucosal melanoma are surgery, radiation, and chemotherapy; but the overall survival remains low.^1^ Cytotoxic T‐lymphocyte associated protein 4 (CLTA‐4) and programmed cell death protein 1 (PD‐1), both inhibitory immune checkpoints commonly seen on activated T cells, have been found to be promising targets for treatment of advanced cancers.^2^ However, the efficacy and response to immunotherapy in mucosal melanoma remains unknown. Herein, we report a case involving a patient, who was a 70‐year‐old male and referred to Taipei Medical University Hospital with confirmed diagnosis of mucosa melanoma over hard plate of mouth. The patient was admitted and subjected to anti‐PD‐1 immunotherapy (pembrolizumab 200 mg every 3 weeks) (Figure 1A). Serial imaging of primary malignant melanoma of the hard palate showed that the tumor size gradually decreased after treatment with pembrolizumab, suggesting partial response/stable disease secondary to continuous immunotherapy (Figure 1B). However, after seventh cycle of treatment, magnetic resonance imaging (MRI) revealed enlarged previous known metastatic lesions and new tumor nodules in brain (Figure 1B). The patient received stereotactic radiation therapy before treatment cycle 14 (Figure 1A). Although the primary metastatic brain lesions were smaller and stationary after radiotherapy, the following brain MRI displayed several hyperdensity masses in the right frontal lobe with perifocal edema and mild mass effect (Figure 1B). Subsequently, patient suffered from infection and respiratory distress and died 2 months after 17^th^ cycle of pembrolizumab therapy.

**FIGURE 1:**
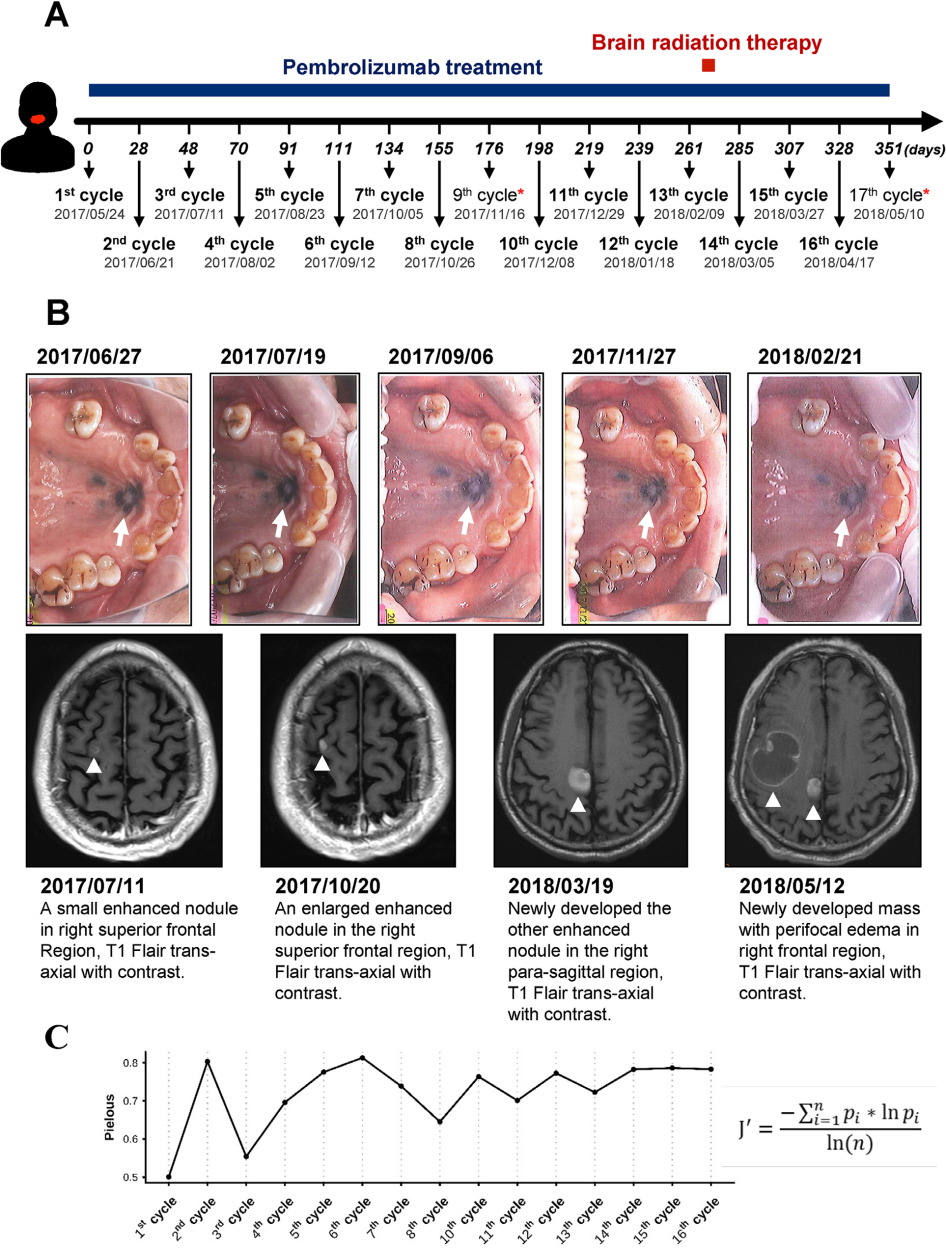
A, Time points of the course of treatment with pembrolizumab and sample collection. Blood samples from the patient were harvested prior to every pembrolizumab injection. Two samples collected at treatment cycle 9 and 17 (labeled with red stars) were removed due to poor RNA quality after nucleic acid extraction. B, Serial imaging of primary malignant melanoma (top panel) and magnetic resonance imaging (MRI) of metastatic brain lesions (bottom panel) of the patient during the immunotherapy with pembrolizumab. C, Temporal dynamics of TCRβ repertoire diversity (Pielou's evenness index) in the patient throughout the treatment course

To monitor changes in patient's circulating T‐cell repertoire at each cycle of immunotherapy, high‐throughput TCRβ sequencing of peripheral blood mononuclear cells (PBMCs), isolated from the patient's peripheral blood, was performed using a rapid amplification of 5′ complementary DNA ends (5′RACE)‐based approach and MiSeq system as previously described.^3^, ^4^ The blood samples were collected from the patient prior to every intravenous injection of pembrolizumab (17 cycles total) over the course of 1 year (Figure 1A). We excluded two samples harvested at treatment cycle 9 and 17 due to poor RNA quality. Thus, a total of 15 TCR repertoire profiles were established. Longitudinal profiling of TCRβ repertoire revealed a rise in TCRβ diversity (Pielou's evenness index) over the course of pembrolizumab therapy (Figure 1C).

To further investigate the temporal dynamics of TCRβ repertoire in the patient during long‐term immunotherapy, the abundance of each TCRβ clonotype was tracked throughout 15 time points. Longitudinal monitoring of clonal abundance revealed a dominant TCRβ clonotype (purple color) in T‐cell repertoire at treatment cycle 1, while other clonotypes exhibited consistent variations throughout the course of treatment (Figure 2A). Overall, rankings of TCRβ repertoire clonotypes did not significantly fluctuate. Nevertheless, the proportion of low‐abundant clonotypes with frequency less than 0.1% (gray color) was significantly increased throughout the treatment (Figure 2A). Within repertoire of TCRβ clonotypes, some high abundance (>1%) clonotypes significantly expanded during the treatment. In particular, three TCRβ clonotypes, two of which were initially low‐abundant (<0.1%), simultaneously expanded more than 10‐fold and became highly abundant (>1%) at cycle 11 and then displayed almost consistent abundance through the last time point of record (Figure 2B). In addition, dramatic expansions of two low‐abundant TCRβ clonotypes at cycle 7 (peak at eighth cycle) and 11 (peak at 12th cycle) were observed (Figure 2B). Since T‐cell‐mediated immune response is usually accompanied by the expansion of polyclonal T cells to tumor antigens,^5^, ^6^ we focused on TCRβ clonotype clusters (groups) rather than single TCRβ clonotype. A clustering method for temporal frequencies of clonotypes was applied to detect low‐abundant clonotypes that possessed similar clonal dynamics.^7^ We herein selected significantly expended TCRβ clonotypes with frequencies reaching top 1% at any time point at least once for the clustering analysis and identified four major groups of clonotypes showing divergent temporal dynamic patterns accordingly. Notably, obvious expansions followed by contractions of two groups of TCRβ clonotypes were detected at treatment cycle 11 (Figure 2C) and 12 (Figure 2D), the time points between new lesion being identified (before treatment cycle 8) and observasion of metastatic brain tumor progression (after treatment cycle 13). Such findings suggested a correlation between partial clonal expansion of TCR repertoire and progression of metastatic tumor lesions, that is, the response of parts of T‐cell clonotypes to the tumor growth, although the overall repertoire diversity was increasing during anti‐PD‐1 treatment. Another two groups of TCRβ clonotypes represented significant polyclonal expansion and contraction with peak at treatment cycle 14 (Figure 2E) and 15 (Figure 2F), both of which coincide in time with postradiation therapy. Such findings showed a strong interventional effect of local radiotherapy on peripheral T‐cell repertoire status of the patient.

**FIGURE 2:**
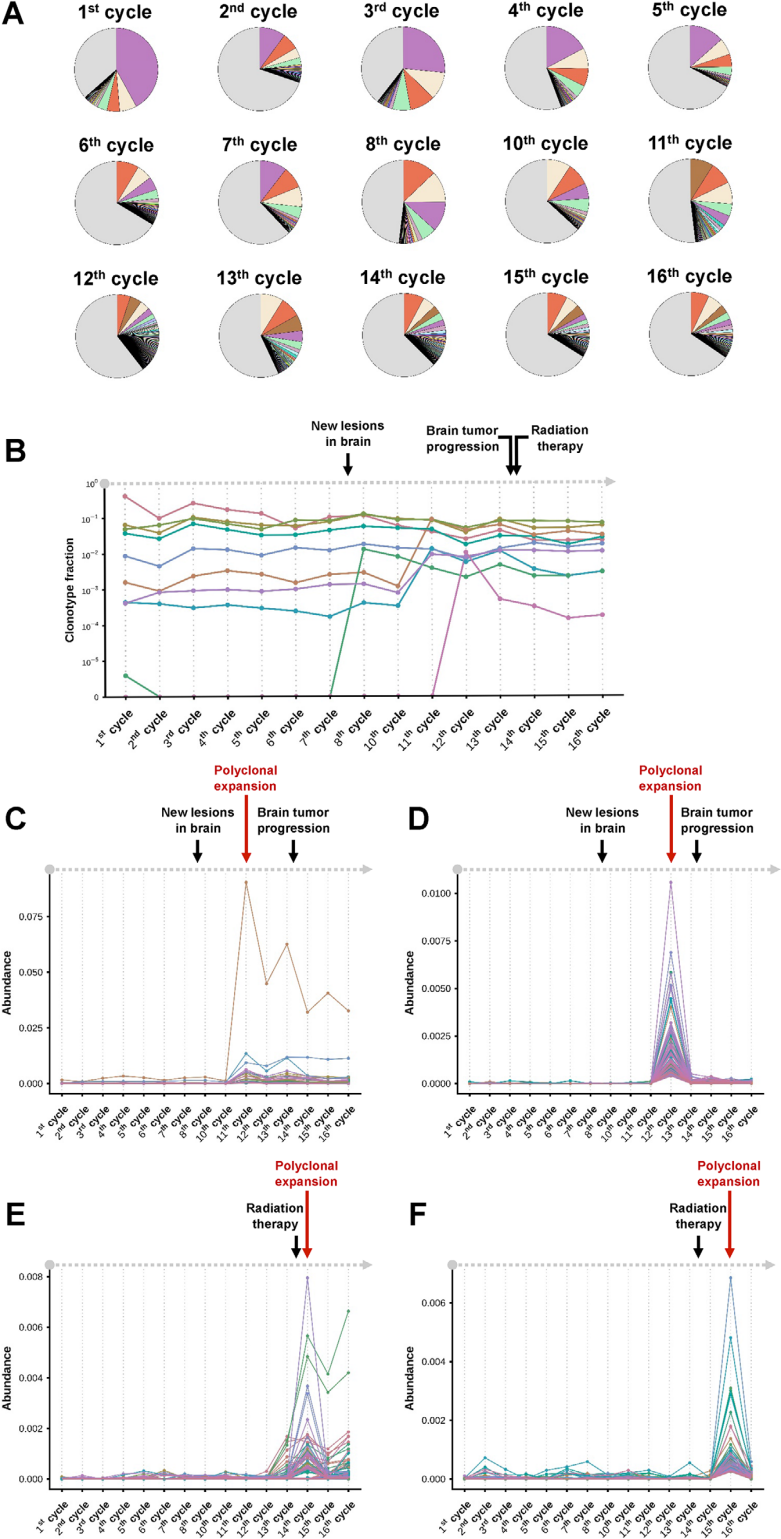
A, Distribution of TCRβ clonotypes of the patient during the pembrolizumab immunotherapy. Only clonotypes with frequency higher than 0.1% at each time point were represented by different colors. Each clonotype was illustrated by identical color across samples. B, Temporal dynamics of TCRβ clonotype abundance in the patient throughout the treatment course. Only abundant clonotypes with frequency greater than 1% at any time point were illustrated. C and D, Two groups of clustered TCRβ clonotypes showing the clonal expansion at time points between clinical observations of new lesions in brain and progression of brain metastasis. E and F, Two groups of clustered TCRβ clonotypes showing the clonal expansion at time points after radiation therapy. Arrows indicated the time points of dominant clonal expansion (red) and the clinical records (black) of the patient. Only abundant clonotypes with frequencies reaching top 1% at any time point at least once were included for clustering analysis

Currently, not much is known about the changes of immune repertoire following long‐term treatment of PD‐1 inhibitor for this type of rare cancer, especially in Asian population. To our knowledge, this is the first report that describes T‐cell repertoire dynamics in a patient diagnosed with mucosal melanoma while receiving long‐term treatment of pembrolizumab. In this study, we carefully monitored TCRβ profiles at 15 time points of 17‐cycle pembrolizumab treatment. Overall, an increased trend of repertoire diversity throughout the whole treatment courses was observed. Such finding is consistent with previous report, which showed a decreased clonality scores of peripheral blood CD8^+^ T‐cell repertoire after anti‐PD‐1 treatment plus brain stereotactic radiation in a melanoma patient.^8^ In addition, clustering of TCRβ clonotypes in this study provided a better resolution to detect low‐abundant clonotypes that possessed similar clonal dynamics as high‐abundant clonotypes. We observed polyclonal expansions within T‐cell repertoire at multiple time points during pembrolizumab treatment. Most of clustered TCRβ clonotypes were initially undetected or low abundant during the early stage of the immunotherapy. These clonotypes showed a clear pattern of clonal expansion followed by contraction at two later time periods where two major clinical events, tumor progression and radiation treatment, could be observed. Our results indicated that the expansion of low‐frequent TCRβ clonotypes within whole TCR repertoire pool might correlate with clinical outcomes of the patient. Consistent with this, association between fluctuation of TCR clonotypes and clinical outcomes during the period of cancer immune therapy has been reported before.^8^, ^9^, ^10^ The clonal expansion of TCR clonotypes might be attributed to antitumoral T‐cell responses to neoantigens derived from tumor cells during cancer progression and treatment.^11^, ^12^ However, more samples and animal models will be helpful to identify pharmacological mechanisms of long‐term combinational immunotherapy in T‐cell repertoire of patients with such a rare melanoma disease.

In conclusion, our study revealed an increase of whole TCRβ repertoire diversity over the anti‐PD‐1 immunotherapy and a large amount of low‐abundant TCRβ clonotypes expanded in the periphery of the patient during disease progression and radiation therapy intervention. Thus, successful identification of the TCR clone expansion, repertoire diversity as well as clustering of low frequency clonotypes might be a useful tool to predict responses of immune checkpoint blockade therapy.

## CONFLICT OF INTEREST

The authors declare no potential conflict of interest.

## ETHICAL APPROVAL

The study was conducted with approval from the Taipei Medical University Joint Institutional Review Board (TMUJIRB) [IRB number: N201904029].

## Supporting information

Supporting Information
