# Supplementary material for: Using T‐cell repertoire profiles as predictor in a primary mucosal melanoma
Source: Clin Transl Med. 2020 Aug 11;10(4):e136. doi: 10.1002/ctm2.136 (PMC7438817; doi:10.1002/ctm2.136)
Supplement: Supplementary file 1 — Supporting Information [file CTM2-10-e136-s001.docx]

**SUPPLEMENTAL METHODS**

**Sample collection and processing**

All peripheral blood samples were collected from the patient of the study at 17 time points prior to each immunotherapy cycle and then subjected to isolation of peripheral blood mononuclear cells (PBMCs) using a density gradient centrifugation method with Histopaque-1077 (Sigma-Aldrich, MO, USA). Subsequently, total RNAs were extracted from patient’s PBMCs using RNeasy mini kit (Qiagen, Hilden, Germany). Due to poor RNA quality, two of samples collected from time points prior to 9^th^ and 17^th^ treatment cycles were excluded from the study. A total number of 15 RNA samples were utilized for following library preparation of T cell receptor sequencing.

**T cell receptor (TCR) sequencing**

Library preparation following high-throughput sequencing of T cell receptor beta chain (TCRβ) for profiling of patient’s T cell repertoire was performed as previously described.[^1^](#_ENREF_1)^,^ [^2^](#_ENREF_2) Full-length cDNA molecules were initially synthesized and enriched from mRNAs of extracted RNA samples using a template-switching reverse transcription polymerase chain reaction (RT-PCR) method with SMARTer PCR cDNA Synthesis Kit (Takara Bio USA, CA, USA). TCRβ fragments with complete VDJ recombinant gene segments were then amplified from total cDNA using template-switching oligo (TSO) sequence as well as T cell receptor beta constant (*TRBC*) genes-specific primers and enriched by size selection, targeting at cDNA molecules ranged from 300 to 1,000 base pairs. Finally, enriched TCRβ fragments were added with Illumina index and adaptor sequences by PCR with Nextera XT Index Kit (Illumina, CA, USA) and purified using AMPure XP (Beckman Coulter, CA, USA). TCRβ libraries from PBMCs collected at different time points were pooled and sequenced by a 2 × 300 pair-end MiSeq v2 system (Illumina, CA, USA).

**T cell repertoire analysis**

Following TCR library preparation and sequencing steps, sequencing raw data in a fastq format was generated and processed as previously described.[^2^](#_ENREF_2) In brief, sequencing reads were subjected to adaptor trimming, quality filtering and then alignment to V, D, and J gene segments of T cell receptor beta locus (*TRB*) gene to extract sequences of complementarity-determining region 3 (CDR3) as TCRβ clonotypes using MiXCR software.[^3^](#_ENREF_3) Abundances of individual TCRβ clonotypes were estimated by calculating frequencies of corresponding CDR3 amino acid sequences.

For measurement of TCRβ repertoire diversity of the patient’s PBMC samples collected from different time points, we calculated Pielou’s evenness index ($J^{'}$):

$$J^{'}=\frac{H^{'}}{H_{max}^{'}}=\frac{-\sum_{i=1}^{n} p_{i}*\ln p_{i}}{\ln(n)}$$

where $H^{'}$ and $H_{max}^{'}$ are derived respectively from actual and theoretical maximal Shannon diversity indices of target TCRβ repertoire, and $n$ and $p_{i}$ represent the total number and abundances of TCRβ clonotypes, respectively.

For clustering of TCRβ clonotypes of the patient’s PBMCs, we performed a network-based analytic approach based on correlation between longitudinal TCRβ clonal abundance profiles across different time points during the immunotherapy. Our clustering method was modified from an original pipeline for identifying cohorts of correlated TCRβ clonotypes in healthy individuals’ TCR repertoires as previously described.[^4^](#_ENREF_4) Our analytic pipeline was as follows: (1) All TCRβ clonotypes which were determined from the patient’s PBMC samples at different time points were pooled together to generate a longitudinal TCRβ repertoire profile. (2) Only TCRβ clonotypes with frequencies reaching top 1% at any time point at least once were selected for clustering. (3) Pearson’s correlation coefficients of pairwise longitudinal profiles of clonal abundance across the patient’s PBMC samples between TCRβ clonotypes were computed and a similarity (correlation coefficient) matrix [-1~1] was generated accordingly. (4) The similarity matrix was then transformed into an adjacency matrix [0, 1] by replacing values of the matrix less than 0.95 or missing by 0. (5) Based on the information of the adjacency matrix, a network was constructed, where vertices represented individual TCRβ clonotypes and edges indicated that dynamic patterns of clonal abundances between two clonotypes were highly correlated or similar. (6) To identify clusters of TCRβ clonotypes with similar clonal dynamics over time, the fast greedy algorithm was applied to the clonotype network. (7) Subnetwork graphs were visualized according to TCRβ clonotypes of each cluster. Majority of the top four clusters with largest number of TCRβ clonotypes were selected and illustrated in Figure 2C-F.

**REFERENCES**

1. Fang H, Yamaguchi R, Liu X, et al. Quantitative T cell repertoire analysis by deep cDNA sequencing of T cell receptor alpha and beta chains using next-generation sequencing (NGS). *Oncoimmunology*. Dec 2014;3(12):e968467.

2. Chang CM, Hsu YW, Wong HS, et al. Characterization of T-Cell Receptor Repertoire in Patients with Rheumatoid Arthritis Receiving Biologic Therapies. *Dis Markers*. 2019;2019:2364943.

3. Bolotin DA, Poslavsky S, Mitrophanov I, et al. MiXCR: software for comprehensive adaptive immunity profiling. *Nat Methods*. May 2015;12(5):380-1.

4. Chu ND, Bi HS, Emerson RO, et al. Longitudinal immunosequencing in healthy people reveals persistent T cell receptors rich in highly public receptors. *BMC Immunol*. Jun 21 2019;20(1):19.
